# Policy Review and Modeling Analysis of Mitigation Measures for Coronavirus Disease Epidemic Control, Health System, and Disease Burden, South Korea

**DOI:** 10.3201/eid2711.203779

**Published:** 2021-11

**Authors:** Hae-Young Kim, In-Hwan Oh, Jacob Lee, Jeong-Yeon Seon, Woo-Hwi Jeon, Jae Seok Park, Sung-Il Nam, Niket Thakkar, Prashanth Selvaraj, Jessica McGillen, Daniel Klein, Scott Braithwaite, Anna Bershteyn, Seung Heon Lee

**Affiliations:** New York University Grossman School of Medicine, New York, NY, USA (H.-Y. Kim, J. McGillen, S. Braithwaite, A. Bershteyn);; Kyung Hee University School of Medicine, Seoul, South Korea (I.-H. Oh, J.-Y. Seon, W.-H. Jeon);; Hallym University Kangnam Hospital, Seoul (J. Lee);; Keimyung University School of Medicine, Daegu, South Korea (J.S. Park, S.-I. Nam);; Institute of Disease Modeling, Seattle, Washington, USA (N. Thakkar, P. Selvaraj, D. Klein);; Korea University Ansan Hospital, Ansan, South Korea (S.H. Lee)

**Keywords:** coronavirus disease, COVID-19, severe acute respiratory syndrome coronavirus 2, SARS-CoV-2, coronaviruses, viruses, respiratory infections, epidemic model, infection control, mathematical modelling, zoonoses, South Korea

## Abstract

We reviewed the timeline of key policies for control of the coronavirus disease epidemic and determined their impact on the epidemic and hospital burden in South Korea. Using a discrete stochastic transmission model, we estimated that multilevel policies, including extensive testing, contact tracing, and quarantine, reduced contact rates by 90% and rapidly decreased the epidemic in Daegu and nationwide during February‒March 2020. Absence of these prompt responses could have resulted in a >10-fold increase in infections, hospitalizations, and deaths by May 15, 2020, relative to the status quo. The model suggests that reallocation of persons who have mild or asymptomatic cases to community treatment centers helped avoid overwhelming hospital capacity and enabled healthcare workers to provide care for more severely and critically ill patients in hospital beds and negative-pressure intensive care units. As small outbreaks continue to occur, contact tracing and maintenance of hospital capacity are needed.

Since the first reported outbreak in Wuhan, China, on December 31, 2019, severe acute respiratory syndrome coronavirus 2 has infected >210 million persons and resulted in nearly 4.4 million deaths worldwide as of August 2021 (*1*). Many countries have responded to the coronavirus disease (COVID-19) pandemic with unprecedented large-scale anticontagion policies, including closure of nonessential businesses and stay-at-home restrictions (*2*). Such policies have had measurable effects on slowing down the epidemic during the early months of the COVID-19 pandemic (*1*–*4*).

In South Korea, the first case of COVID-19 was reported on January 20, 2020, and an additional 27 cases were confirmed by February 10. All confirmed case-patients either had international travel histories linked to the cities with confirmed cases or were the contacts of index case-patients. Many of these early cases were likely linked to travel between Wuhan and South Korea during the Lunar New Year holiday on January 24‒28, 2020. However, on February 18, a woman in Daegu, the epicenter of the initial COVID-19 outbreak in South Korea, was the first case-patient who had no international travel history or contact with another index case-patient. Epidemiologic surveillance showed that she attended a large Shincheonji Church meeting before her diagnosis. Subsequently, >2,500 cases (62% of all confirmed cases in Daegu) were confirmed positive and epidemiologically linked to this church. The rapid surge in cases quickly overwhelmed all available hospital beds and intensive care unit (ICU) capacity in Daegu (*5*,*6*).

In response to the rapid surge, the government of South Korea implemented intensive policies for testing, contact tracing, and quarantining of all close and potential contacts of index cases, and social distancing (*7*). We review the timeline of key policies and practices implemented for COVID-19 epidemic control during the early 2020 epidemic in South Korea. We then used a stochastic transmission model to retrospectively evaluate the probable impact of these policies and practices on the epidemic control. Our findings offer lessons for future health system planning and epidemic control during an initial outbreak of a respiratory disease.

## Methods

### Review of Country-Level Responses to COVID-19 Outbreak

We reviewed and summarized the key policies for COVID-19 epidemic control in South Korea during January 1‒May 15, 2020. Our review used the World Health Organization (WHO) operational guidelines for COVID-19 strategic preparedness and response plan to categorize components of the response (*8*). The guidelines focus on 9 major pillars: 1) country-level coordination, planning, and monitoring; 2) risk communication and community engagement; 3) surveillance, rapid-response teams, and case investigation; 4) points of entry, international travel, and transport; 5) national laboratories; 6) infection prevention and control; 7) case management; 8) operational support and logistics; and 9) maintaining essential health services and systems. The details of the epidemic, policies, and health system use were collected from the official site of the Korea Center for Disease Control and Prevention (KCDC) and Daegu Disaster Management Headquarters (*5*,*9*), which were made public daily. We provide major policies at each governmental level, facility level, congregate setting, and household/personal level in chronological order (Table 1; Appendix Figure).

**Table 1:** Summary of key policies and practices for COVID-19 epidemic control and responses, South Korea, January 1‒May 15, 2020 (*8*)*

Pillars	Category	Description
Country-level coordination, planning, and monitoring	School closure	Postpone nationwide school opening after winter break.
	Workplace closure	Voluntary participation of employers to enable work from home and closure of nonessential business.
	Public events cancellation	Recommended cancelling religious services and large in-person gatherings.
	Public transport	All public transport systems remained open. The Seoul Metro system made it mandatory to wear a mask when riding the subway.
	Restriction of internal movement	Daegu City and Gyungbuk Province were designated as special management regions for COVID-19 on February 23, but no city-wide lockdown or stay-at-home restrictions were imposed. However, many persons voluntarily refrained from movement and gatherings.
	Promotion of personal protective measures	Promoted mask wearing in all public locations and frequent handwashing.
	National hotline for case reporting and testing	Opened a national hotline (#1339) for anyone who had fever, cough, or difficulty breathing to report and provided tests at screening stations.
Risk communication and community engagement	Rapid policy updates	The KCDC held daily briefings to provide status updates and policy guidance.
	Public disclosure of the trajectories of confirmed cases and alert system	The government publicly shared the trajectories of confirmed cases and sent alerts to those living in the areas where the cases were confirmed.
Surveillance, rapid-response teams, and case investigation	Contact tracing of direct contacts	Contact tracers called and traced all direct contacts of confirmed cases, where direct contacts were defined as anyone exposed to a confirmed COVID-19 case from 2 d (or 1 d since April 3) before symptom onset (or confirmed testing date for asymptomatic cases) to the last day of quarantine of the index case. For example, >99% of Shincheonji Church members were traced. A mobile app was launched on March 29 to trace the trajectories of confirmed cases in 10 min by linking the data from 28 related national institutions.
	Self-quarantine of contacts	All close contacts of confirmed case were required to self-quarantine for at least 2 weeks.
	Active monitoring and wide testing of potential contacts	Anyone who had overlapping trajectories with cases was alerted and offered free testing.
Points of entry, international travel, and transport	Self-quarantine and monitoring of in-bound travelers	At entry screening, travelers entering South Korea (both Korean and foreign nationals) were tested if they have fever or respiratory symptom. Korean nationals or foreign nationals on long-term visas were asked to self-quarantine since March 19. Foreign nationals on a short-term visa were quarantined at a temporary quarantine facility. A self-health check app has been used to monitor the health of in-bound travelers at least once daily for the 14 d following their arrival date.
	Travel history monitoring at hospitals	Tracing and alarm system (DUR/ITS) connected to the national insurance system filtered and screened persons who had an international travel history.
National laboratories	Rapid increase in testing capacity	Rapid set-up for RT-PCR for COVID-19 diagnosis in late January. RT-PCR became available in 46 laboratories by February 7, and 77 laboratories by February 20 with a testing capacity for 13,000 tests/day by the end of March.
Infection prevention and control	Designated hospitals for nonrespiratory medical visits only	From February 21, national safe hospitals were designated for medical visits related to nonrespiratory symptoms to separate these patients from potential COVID-19 patients.
	Preparation of negative pressure beds in hospitals	50 beds in NPIRs were added in Daegu on February 21 and additional 120 beds with NPIR on March 17.
	Preparation of personal protective equipment in hospitals	Healthcare workers were prioritized to receive public mask supply.
	Screening residents in long-term facilities (i.e., nursing homes)	Thorough investigation for unexplained pneumonia and COVID-19 testing were performed among the residents in 450 nursing homes on March 5.
Case management	Isolation of confirmed cases	Asymptomatic case-patients were isolated in designated CTCs starting on March 2. Symptomatic case-patients who had moderate and severe symptoms were isolated in hospitals. Anyone who violates the self-quarantine rule was to be charged a penalty of up to $10,000 or 1-y imprisonment.
	Triage of severe cases	Introduced a COVID-19 triage system based on disease severity (grades 1 to 4).
	Reallocation of hospital and ICU beds	Prioritization and reallocation of hospital and ICU beds for critically ill patients.
Operational support and logistics	Staff surge capacity and deployment at hospitals and airports	Special health workforce of ≈2,000, including 750 public health doctors, 172 specialists, 346 physicians, and 728 nurses were recruited and dispatched to hospitals in Daegu and Gyeongsangbuk Province to support COVID-19 case management as of March 9.
		Approximately 300 military doctors and nursing officers had supported screening and quarantine at airports and seaports until April 27.
	Face mask supply	The government rapidly increased the supply chain to produce 12 million disposable masks per day and set a mask rationing system to secure mask supplies and meet demands. Each person was permitted to buy 2 masks/week on an assigned weekday based on the last digit of the person’s birth year.
	Preparation of public health centers, hospital beds, and medical equipment	Total number of hospital beds with NIPRs was expanded from 198 to 1,077 beds by February 22.
	Screening stations	523 screening clinics were launched nationwide including drive-thru and walk-thru screening stations that had reversible negative/positive pressure booths.
Maintaining essential health services and systems	Triage and separation of non-COVID-19 patients	Daegu Dongsan Hospital and Daegu medical centers were designated as COVID-19 central hospitals where a massive surge developed on February 21; admitted patients with other disease were evacuated to other hospitals on February 23.
		National safe hospitals were designated where only nonrespiratory patients could seek medical care.

*COVID-19, coronavirus disease; CTC, community treatment center; DUR/ITS, drug utilization review/international traveler system; ICU, intensive care unit; KCDC, Korea Centers for Disease Control and Prevention; NPIRs, negative-pressure isolation rooms; RT-PCR, reverse transcription PCR.

### Mathematical Model

We adapted an existing stochastic, discrete-time compartment model of community transmission of severe acute respiratory syndrome coronavirus 2 (*10*) to simulate the COVID-19 epidemic in South Korea (Appendix) . The model represents persons who are susceptible (S), exposed (E), infectious (I), or removed/recovered (R). We assumed that initial infections were imported through international travelers.

After the daily cases peaked at 813/day on March 1, the daily cases decreased below 100/day, and >99% of all Shincheonji Church members in Daegu were successfully traced and tested by mid-March. By March 18, there were 8,413 confirmed cases, 270,888 confirmed negative tests results, and 16,346 tests in progress, yielding a positive test rate of 3.0% and a case-fatality rate of 1.00%. Given that testing was widely conducted with intensive efforts for contact tracing during the initial outbreak, we assumed that the case-fatality rate is not far above the infection-fatality rate (IFR). To be conservative, we assumed that the true IFR would be slightly lower by a factor of 5%, resulting in the overall IFR estimate of 0.95.

### Model Assumptions and Calibration

As of May 15, there were 10,991 confirmed cases, 695,854 confirmed negative test results, 19,875 in progress for test results, and 260 cumulative deaths. According to the data published by KCDC and Daegu Disaster Management Headquarters, 69.1% of all case-patients were asymptomatic or mildly symptomatic, 22.4% had mild symptoms, 10.0% had severe symptoms leading to hospitalization, and 3.6% had critical illness requiring ICU admission (*5*).

The government of South Korea encouraged all case-patients, including mild symptomatic and asymptomatic case-patients, to be hospitalized to prevent community transmission, yielding a high case-hospitalization rate. Although 49.4% of all case-patients were hospitalized before March 2, a total of 82.6% of all infected case-patients were either hospitalized or admitted to community treatment centers (CTCs) after these centers were established to provide care for asymptomatic or mildly ill patients after March 2, 2020. We assumed that the average time from infection to symptom onset was 5.1 days (*11*) and from symptom onset to hospitalization was 4 (range 0–11) days (*12*–*14*) (Table 2). Our previous study used the claims made in the National Health Insurance System (NHIS) (*15*), a mandatory health insurance system covering 96.6% of the entire population of South Korea. On the basis of those data and data from the literature, we assumed that the average length of stay at hospitals among non‒ICU-admitted patients would be 21 (7.2–32.6) days and that for case-patients quarantined at CTCs would be 16 (7–20) days (*15*–*18*).

**Table 2:** Input parameters for COVID-19 transmission compartmental model, South Korea*

Characteristic	Baseline value	Range†	Reference
Hospitalizations for all confirmed cases by disease severity before March 2, %	49.4		(*5*)
Asymptomatic or mild symptomatic cases	17.0		(*5*)
Mild symptomatic cases	22.4		(*5*)
Severely ill cases	6.4		(*5*)
Critically ill cases	3.6		(*5*)
Hospitalizations for all confirmed cases by disease severity after March 2, %	82.6		(*5**,**9*)
Asymptomatic or mild symptomatic cases (admitted to CTCs)	38.2		(*5**,**9*)
Asymptomatic or mild symptomatic cases (admitted to hospitals)	12.0		(*5**,**9*)
Mild symptomatic cases	22.4		(*5**,**9*)
Severe symptomatic cases	6.4		(*5**,**9*)
Critically ill cases	3.6		(*5**,**9*)
Proportion admitted to the ICU among hospitalized patients	8.1		(*5**,**9*)
Time to outcome, d			
Time to symptom onsets	5.1	4.5‒5.8	(*11*)
Time from symptom onset to hospitalization	4.0	0‒11	(*12**‒**14*)
Time from symptom onset to ICU hospitalization	7.0	6‒8	(*12**‒**14*)
Time from symptom onset to death	17.0	0‒27	(*14*)
Length of stay at CTC	16.0	7‒20	(*9**,**18*)
Length of stays at hospital without ICU admission	20.9	7.2‒34.6	(*15**‒**17*)
ICU length of stay among survivors	30.0	11.6‒47.2	(*5**,**15**,**16*)
ICU length of stay among nonsurvivors	10.0	0 ‒13	(*5**,**15**,**16*)
Case-fatality rate for critically ill patients, %	60.0		(*5**,**9*)

*COVID-19, coronavirus disease; CTC, community treatment center; ICU, intensive care unit. †Range was used for sensitivity analysis where available. For parameters that were calculated as proportions, baseline values were used as fixed values.

Critically ill patients were assumed to be first admitted to non-ICU hospital beds for 3 days, then transferred to the ICUs for 30 (range 11.6–47.2) days before returning to non-ICU hospital beds for another 5 days (*5*,*15*,*16*). On the basis of KCDC data, we assumed that 60% of the critically ill patients would die and have an average length of time in the ICU of 10 (range 0–13) days before death (*5*,*15*,*16*).

We calibrated the susceptible-exposed-infectious-removed (SEIR) model to the data for confirmed COVID-19 case-patients, hospital census, CTC census, ICU census, and deaths as reported by KCDC and Daegu Disaster Management Headquarters during February 1‒May 15, 2020 (*5*,*9*). We estimated the basic reproduction number (R_0_) at the beginning of the epidemic in South Korea and the effective reproduction number (R_e_) after the first epidemic peak in early March 2020. We adjusted the estimated R_e_ to minimize the sum of squared residuals between the data and the corresponding model outputs after the epidemic started decreasing in late February. To enable stochasticity in transmission, we applied a log-normal stochastic process with an SD of 0.722, a value determined on the basis of fitting this model to the 2018–19 influenza season for Seattle, Washington, USA (*10*). We implemented the model in Python version 3.7 (https://www.python.org) and analyzed and graphed outputs by using R version 3.6.1 https://www.r-project.org). Ethics approval was not required because the study was based on a simulated cohort of patients and used publicly available epidemiologic data.

### Model Scenarios for Impact of Mitigation Measures

Rapid testing and effective contact tracing of index cases enable health authorities to test and quickly quarantine infectious persons and isolate the contacts of index case-patients from the susceptible population, reducing the number of infectious persons in the population and thus preventing onward transmissions. Several studies, including 2 meta-analyses of respiratory diseases caused by coronaviruses, showed that social distancing and mask-wearing reduce viral transmission among contacts (*19*,*20*). In our SEIR model, we assumed that social distancing and mask-wearing would reduce transmissibility or contact rates for infectious persons. Given that all mitigation measures and interventions, including contact tracing and testing, social distancing, and mask-wearing, had simultaneously occurred, we did not separately model and measure the effects of individual interventions but estimated the overall impact of combined interventions.

We measured outcomes of the epidemic (infections, cases, and deaths) and health system burden (hospital census, CTC census, and ICU census) by May 15, 2020, in South Korea associated with the actual response and compared them with hypothetical, less intensive mitigation efforts. Specifically, we considered 2 scenarios where R_e_ was estimated to be reduced by 50% of the initial R_0_ by February 28, then would remain at 50% (scenario 1) or 70% (scenario 2) of the initial R_0_ after February 28. We also conducted sensitivity analysis by varying the key parameter values affecting health system burden (Appendix Figure 2).

## Results

We present a summary of key policies and practices for COVID-19 response and control in South Korea. This summary was conducted according to WHO guidelines (Table 1; Appendix Figure 1).

### Key Policies and Practices

#### Country-Level Coordination, Planning, and Monitoring

A special COVID-19 task force was organized on January 3, 2020. As soon as the first COVID-19 case was confirmed, the government of South Korea promptly declared its political commitment on January 22 to prepare a response to COVID-19 in advance of the Lunar New Year holidays (*21*).

#### Risk Communication and Community Engagement

The government raised the alarm level in the 4-level national crisis management system (blue, yellow, orange, red) from yellow (stage 2) to orange (stage 3) on January 27 and to red (stage 4) on February 23, after the WHO Public Health Emergency of International Concern announcement on January 30. The KCDC held daily briefings to provide status updates and policy guidance to the public.

#### Surveillance, Rapid-Response Teams, and Case Investigations

We implemented intensive contact investigation and quarantine for all potential contacts of index case-patients (*5*,*21*,*22*). Epidemiologic Intelligence Service officers rapidly traced the contacts of every confirmed index case-patient by using cell phones and novel mobile applications (*23*). During January 20‒March 27, 2020, the number of index case-patients traced was 5,706, and the number of contacts traced was 59,073, yielding a ratio of contacts traced/index case patient of 10.4 (*24*). The contacts who were successfully traced were monitored for an average of 9.9 days (*24*).

#### Points of Entry, International Travel, and Transport

After March 19, all in-bound passengers received health screenings at airport immigration checkpoints (*9*,*25*,*26*). These screenings were performed to identify new case-patients coming into South Korea.

#### National Laboratories

The Academy of Korean Laboratory Medicine developed reverse transcription PCR (RT-PCR)‒based COVID-19 diagnostic kits, which were rapidly approved by the Korean Food and Drug Administration and distributed to 18 public laboratory centers on January 31. Rapid approval by the Korean Food and Drug Administration was possible because the government had established a system that enables emergency-use authorization in vitro diagnostics after the outbreak of Middle East respiratory syndrome (MERS) during 2015 (*27*).

#### Infection Prevention and Control at Hospitals

All hospitals and public health centers set up COVID-19 screening clinics after the first case was confirmed in South Korea. The transmission risk among healthcare workers was low; only 241 (2.4%) of all confirmed cases were healthcare workers as of April 5 (*9*).

##### Congregate Settings

On March 25, the government completed a full screening of high-risk congregate facilities, as well as nursing homes (*28*,*29*). This screening showed a positivity rate of 0.7% (224/32,990) in Daegu.

##### Social Distancing

The government announced a nationwide social distancing campaign for 2 weeks starting March 22, 2020. This campaign included staying home except for essential travel, limiting social gatherings, working from home whenever possible, and keeping 6 feet of distancing from others outside the home (*30*). In addition, after the mass outbreak occurred in Daegu in February, persons voluntarily reduced mobility and increased social distancing (e.g., the total number of riders taking the Seoul subway decreased to half of its previous total) (*31*). The government later established guidelines for implementing 3 levels of social distancing based on the number of confirmed cases in the local area (*25*).

##### Use of Face Masks

Since 2014, a yellow dust storm that originated in the deserts of Mongolia and northern China during the spring has been a public health issue in South Korea, and persons were advised to wear a face mask outdoors to avoid inhaling particulate matter. In addition, because of an outbreak of MERS during 2015 that resulted in 186 cases and 38 deaths, public acceptance of wearing a mask was high in the event of respiratory disease outbreak. Wearing a face mask in public areas was regarded as a sign of thoughtfulness and modesty to prevent transmission to others (*32*). The 2 surveys conducted in late February and mid-March 2020 showed that 63% (*33*) and 94% (*34*) of persons in South Korea reported always wearing face masks when they were outside.

#### Case Management

Several CTCs were established on March 2 to quarantine and monitor asymptomatic and mild symptomatic case-patients and to enable reallocation of hospital beds in Daegu, when the total number of isolated patients, including self-quarantined cases at home waiting for admission (4,159), exceeded the number of available hospital isolation beds. Shortly afterward, CTCs were implemented nationwide (*18*). In addition, Daegu Dongsan Hospital and Daegu Medical Center were designated as COVID-19 central hospitals for effective COVID-19 case management (*35*).

#### Operational Support and Logistics in Hospitals

South Korea had the second-highest number of hospital beds per capita (12.3 beds/1,000 population) worldwide during 2019 (*36*). The number of negative-pressure beds increased to 1,077 by February 22 during the early part of the outbreak (*9*). In addition, to accommodate the rapid surge of COVID-19 patients, most tertiary hospitals constructed and renovated their isolation rooms with airborne infection isolation using common outlet duct systems or mobile negative-air machines (*37*).

##### Human Resources

Public health and army doctors dispatched as a substitute for their obligatory military service. These doctors were the main workforces, in addition to thousands of medical volunteers.

#### Maintaining Essential Health Services and Systems

Some private and public hospitals were designated as COVID-19 central hospitals. This designation was conducted to care only for patients with confirmed COVID-19.

### Estimated Impacts of Policy and Interventions

The epidemic rapidly increased in the early phase, and the number of new daily cases peaked at 656 on February 29 (Appendix Figure 1). However, new daily cases declined in March and reached fewer than 100 daily confirmed cases after April 2. The reported hospital census peaked on March 14 at ≈3,600 cases and the CTC census on March 15 at 3,025 cases (Figure). The ICU census reached its peak in mid-March at ≈160. Given the limited capacity of ≈3,600 available hospital beds for isolation and 300 ICU beds with negative pressure in South Korea (*6*,*16*), a delay in governmental response for epidemic control is likely to have caused the epidemic to exceed the existing hospital capacity nationwide.

**Figure:**
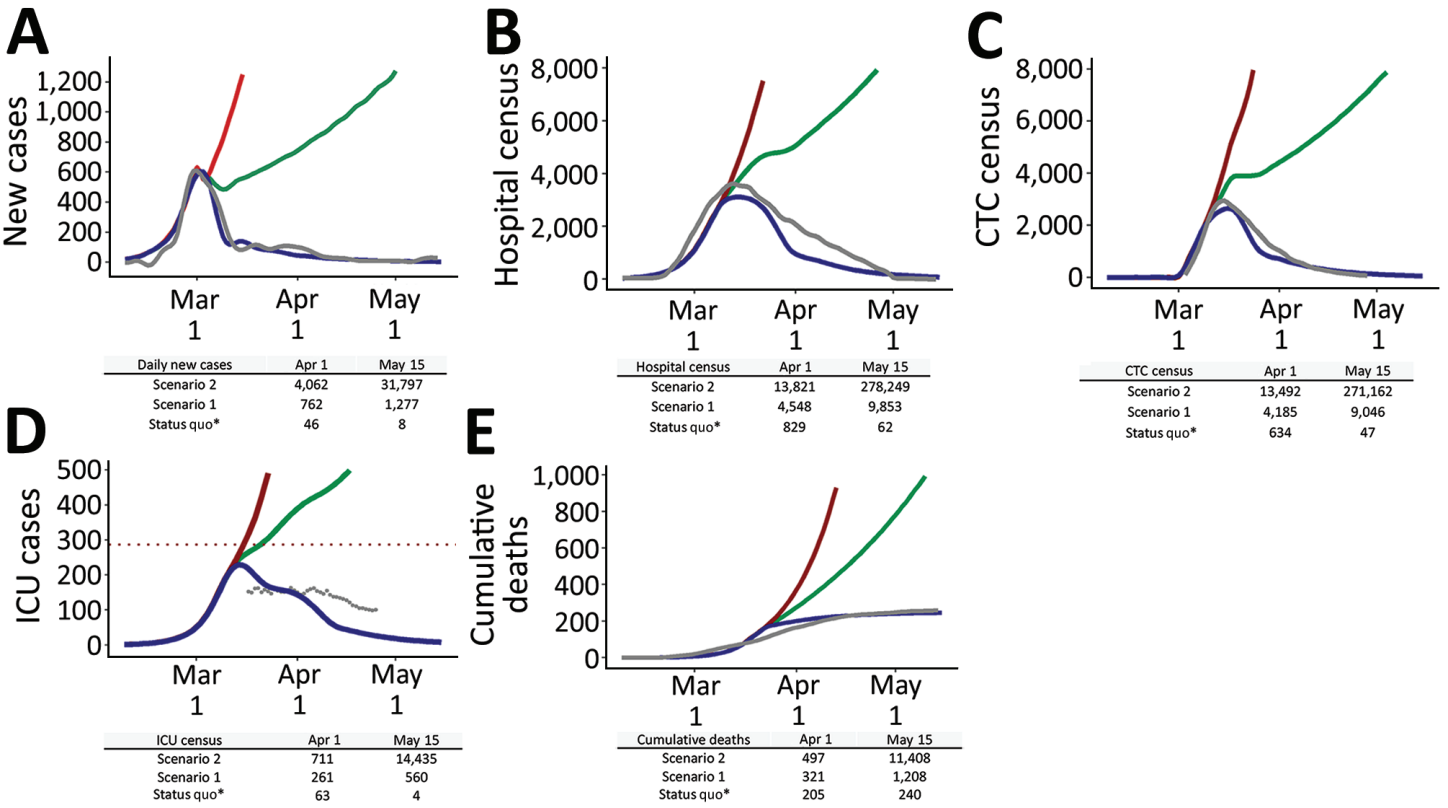
Estimated and confirmed numbers for coronavirus disease, South Korea, 2020. A) New daily cases; B) hospital census; C) CTC census; D) ICU census; E) cumulative deaths. Gray lines indicate observed data (*5*,*6*,*9*,*16*). Blue lines indicate estimated numbers with 35%, 50%, and 90% reductions in the basic reproduction number by February 26, February 28, and March 2, respectively, as the status quo. Additional scenarios are shown where R_0_ was assumed to be reduced by 70% (scenario 1, green line) or stayed the same at 50% (scenario 2, red line) after February 28. CTC, community treatment center; ICU, intensive care unit; R_0_, basic reproduction number.

The SEIR model estimated that R_0_ was 3.24 at the beginning of the outbreak but decreased by 35% as of February 26, 50% as of February 28, and 90% as of March 2 (Figure). Such a reduction can be attributed to the combination of different mitigation efforts and individual practices as described earlier in this report, including contact tracing of ≈99% contacts in the Shincheonji Church outbreak in Daegu and isolation of contacts at hospitals or CTC, social distancing and voluntary reductions in population mobility, near-universal mask wearing in public, and widespread testing.

The SEIR model estimated that the number of new daily cases would have exceeded 750 by April 1 and resulted in ≈27,000 cumulative infections if R_0_ had been reduced only by 70%, which showed ≈25% lower composite effects of mitigation measures on reducing contact rates and transmissibility compared with the status quo (scenario 1) (Figure). R_0_ would have remained at ≈1, sustaining the continued epidemic growth and outbreak clusters. By May 15, the cumulative infections would have reached 82,000 and the hospital census would have reached 9,900, which is ≈3 times higher than the total hospital beds available for isolation in South Korea. Cumulative deaths would have exceeded 1,200, which is >5-fold increase over the number of cumulative deaths observed as of May 15. This result would have prompted nationwide stage 2.5 social distancing measures and restrictions in which persons are advised to stay at home, and private or public gatherings of >50 persons indoors are prohibited (*31*).

If R_0_ had been reduced by only 50% after February 28, the epidemic would have reached 4,000 new daily cases and 83,900 cumulative infections by April 1 and 31,800 new daily cases and >1.7 million cumulative infections by May 15 (scenario 2) (Figure). R_0_ would have reached ≈1.6, exponentially increasing and doubling the cases by 7.9 days nationwide. This result would have prompted nationwide stage 3 social distancing measures and restrictions, the highest level of restriction, in which persons are advised to strictly stay at home, all nonessential businesses and in-person schooling are closed, and private or public gatherings of >10 persons indoors are prohibited (*31*).

## Discussion

Our SEIR model showed that swift and comprehensive coordination and preparation of the government in response to the spring 2020 COVID-19 outbreak achieved rapid epidemic control in Daegu and nationwide by reducing R_0_ by 90% through various interventions, including widespread testing, contact tracing, and quarantine without strict lockdown of the city or stay-at-home orders. Without these prompt multilevel responses, the epidemic could have led to a >10-fold increase in cumulative infections and deaths by May 15. The model also estimated that a delay in the government’s response or an absence of rapid triage of mild symptomatic case-patients from hospitals to CTCs would have exceeded the hospital system capacity for hospital beds and negative- pressure rooms and potentially resulted in more deaths by overburdening the health system.

Several key factors contributed to slowing down the epidemic without a citywide or nationwide lockdown. The government intensively used an active tracing system that consisted of location tracking, card transactions, closed-circuit television recording, and a digital tracing mobile application to trace not only close contacts but also all potential contacts and offer testing to them. This system was possible because of the rapid set-up of RT-PCR capacity to perform 15,000–20,000 tests/day by early February and publicly disclosing the trajectories of confirmed COVID-19 case-patients so that anyone who might have contacted confirmed case-patients could self-identify and receive testing. Many of these lessons were learned from the MERS outbreak in South Korea during 2015.

A delayed response would have resulted in a surge of case-patients that would have overwhelmed the available hospital capacity nationwide. In Daegu, where 75% of the confirmed cases were located, the ICU census already exceeded the available ICU bed capacity (≈60) in public hospitals by late February, and an additional 50–60 critically ill patients were transferred to hospitals outside Daegu (*5*). In addition, establishing CTCs to isolate and manage asymptomatic case-patients was critical to effectively control further community transmission and to reduce burden on the hospital system (*38*).

Since mid-May 2020, South Korea has experienced several clustered outbreaks, including 1 at the Itaewon night club and others at multiple logistics centers (*39*). These outbreaks suggest that community transmission can quickly escalate and could lead to a large surge in cases after relaxing social distancing polices. In preparation for potential community outbreaks and surges in cases, the government arranged an additional 1,077 hospital beds, negative-pressure areas, and 300 ICU beds nationwide. It also eased the hospital discharge criteria for a shorter turnover time of hospital beds so that symptomatic patients could be discharged if their clinical symptoms improved without fever for 10 days after symptom onset, or if RT-PCR results were negative for >24 hours after the confirmed diagnosis (*9*). Monitoring and contact tracing continued to be central to the COVID-19 response in South Korea, especially for high-risk groups (*40*), and hospital bed capacity was maintained at designated COVID-19 management facilities in the event of further outbreaks.

The first limitation of our study is that we have not explicitly modeled quarantine or contact tracing and did not estimate the effects of individual interventions. Instead, we assumed that the combination of all interventions and policies reduced overall transmission rates in the population. Second, our SEIR compartment model did not capture any spatial networks among different cities in South Korea. Third, data that informed input parameters for modeling are subject to uncertainties and should be validated with further clinical data.

In summary, our model estimates that South Korea reduced contact rates by 90% through various interventions without strict lockdown of the city or stay-at-home restrictions. At the same time, allocation and management of mild and moderate symptomatic case-patients helped to avoid overburdening the hospital system. However, continuous monitoring, contact tracing, securing hospital and isolating beds, and social distancing will remain critical as long as COVID-19 outbreaks remain a public health threat.

Appendix Additional information on policy review and modeling analysis of mitigation measures for coronavirus disease epidemic control, health system, and disease burden, South Korea.
