# Supplementary material for: Policy Review and Modeling Analysis of Mitigation Measures for Coronavirus Disease Epidemic Control, Health System, and Disease Burden, South Korea
Source: Emerg Infect Dis. 2021 Nov;27(11):2753–60. doi: 10.3201/eid2711.203779 (PMC8544960; doi:10.3201/eid2711.203779)
Supplement: Appendix — Additional information on policy review and modeling analysis of mitigation measures for coronavirus disease epidemic control, health system, and disease burden, South Korea. [file 20-3779-Techapp-s1.pdf]

Article DOI: https://doi.org/10.301/eid2711.203779

Policy Review and Modeling Analysis of Mitigation Measures for Coronavirus Disease Epidemic Control, Health System, and Disease Burden, South Korea

Appendix

Susceptible-Exposed-Infectious-Removed Transmission Model

We adapted an existing stochastic, discrete-time, compartment model of community transmission of severe acute respiratory syndrome coronavirus 2 based on the report by Wu et al. (*1*) and that described by Thakkar et al. (*2*). The model structures can be described as the following:

$$S_t = S_{t-1} - \beta S_{t-1}(I_{t-1} + z_{t-1})\varepsilon_t$$

$$E_t = \beta S_{t-1}(I_{t-1} + z_{t-1})\varepsilon_t + \left(1 - \frac{1}{D_e}\right)E_{t-1}$$

$$I_t = \frac{1}{D_e}E_{t-1} + \left(1 - \frac{1}{D_i}\right)I_{t-1}$$

$$R_t = \frac{1}{D_I}I_{t-1}$$

$$C_t \sim Binomial(I_t, p)$$

where D_e is the latency period (4 days), D_i is the infectious duration (8 days), β is the attack rate at the beginning of the outbreak, and z_t is the number of importations on day t. In South Korea, a total of 16 cases were confirmed as international importations by February 10, 2020, including 11 cases associated with Wuhan, China, and 5 cases linked to other global importations (*3*). Therefore, z_t was set to 11 on January 10 for the importations linked to Wuhan, and to 5 on January 27 for the cases linked to other global importations. In the model, transmission is a log-normal stochastic process with Var[ln ε_t] = σ_ε^2, and case detection is a binomial process with reporting rate of p. We have set σ_ε to 0.722, a value determined from the 2018–19 influenza season for Seattle, Washington, USA.

Sensitivity Analysis for Health System Burden

We have simulated the model projections by varying the key parameters for the range, as shown in Table 2, which affect length of stay at non–intensive care units (hospital beds), community treatment centers, and intensive care units.

References

1. Wu JT, Leung K, Leung GM. Nowcasting and forecasting the potential domestic and international spread of the 2019-nCoV outbreak originating in Wuhan, China: a modelling study. Lancet. 2020;395:689–97. PubMed https://doi.org/10.1016/S0140-6736(20)30260-9

2. Thakkar N, Klein D, Selvaraj P, Famulare M. COVID in New York City: a model-based perspective, 2020 [cited 2021 Jun 26]. https://iazpvnewgrp01.blob.core.windows.net/source/archived/COVID_Modeling_NYC.pdf

3. Hyun J, Lee JH, Park Y, Jung EK. Interim epidemiological and clinical characteristics of COVID-19 28 cases in South Korea. Public Health Wkly Rep. 2020;13:472–4 [cited 2021 Jul 28]. https://kdca.go.kr/board/board.es?mid=a20602010000&bid=0034&list_no=366372&act=view

4. Daegu Disaster Management Headquarters. COVID-19 daily briefing in Daegu metropolitan City, 2020 [cited 2020 Jun 30]. http://www.daegu.go.kr/dgcontent/index.do?menu_id=00936590

5. Central Department of Mental Health. Cases in Korea by city/province, 2020 [cited 2020 Aug 12]. http://ncov.mohw.go.kr/en/bdBoardList.do?brdId=16&brdGubun=162&dataGubun=&ncvContSeq=&contSeq=&board_id=&gubun=

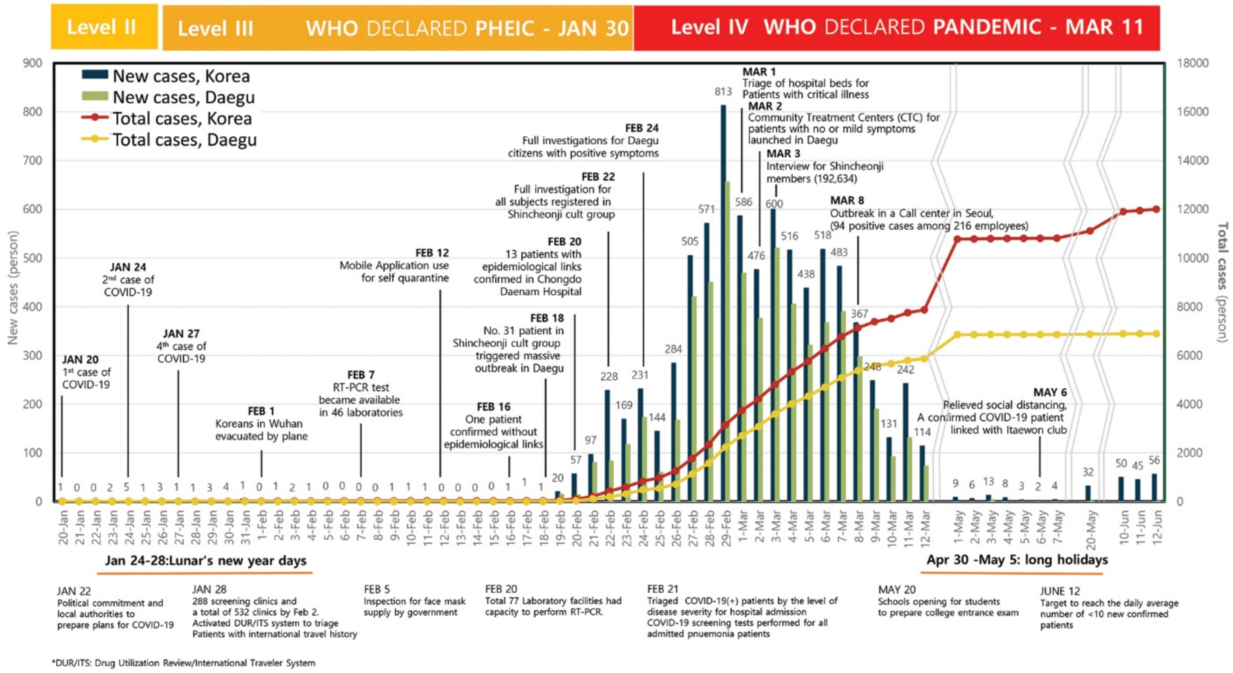

Appendix Figure 1. Timeline of key policies and the number of new and total cases of coronavirus disease in Daegu (*4*) and South Korea (*5*). COVID-19, coronavirus disease; CTC, community treatment center; DUR/ITS, drug utilization review/international traveler system; PHEIC, public health emergency of international concern; RT-PCR, reverse transcription PCR; WHO, World Health Organization.

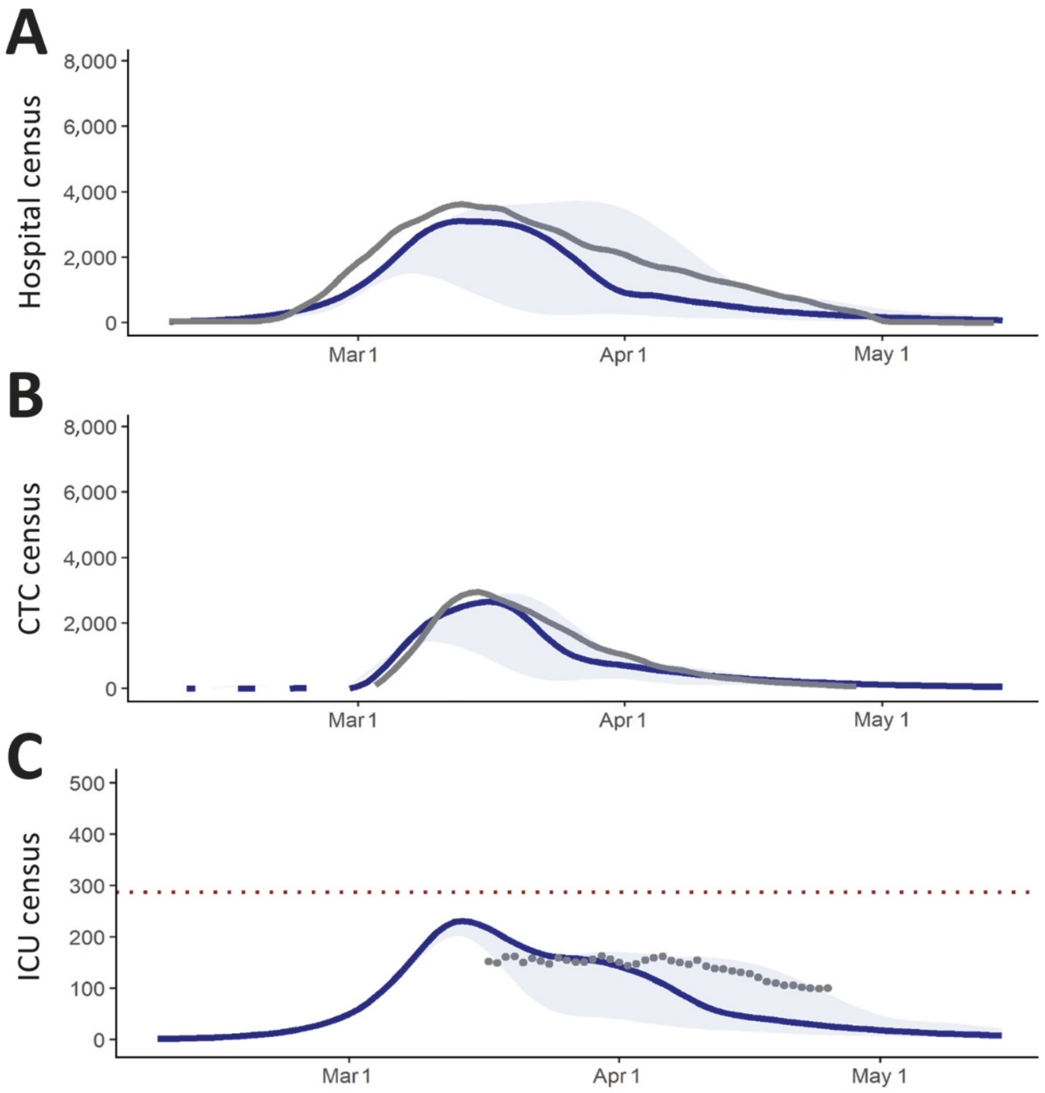

Appendix Figure 2. Estimated numbers of coronavirus diseases cases by (A) hospital census, (B) CTC census, and (C) ICU census in sensitivity analysis. Gray lines and points indicate observed data for South Korea. Blue lines indicate estimated numbers as the status quo. Shaded blue area is bounded by projections at the minimum and maximum of the key parameters affecting the length of stay at non-ICU hospital, CTC, and ICU, respectively. CTC, community treatment center; ICU, intensive care unit.
